# Immune-related myocarditis characterized by malignant arrhythmia caused by conduction system involvement: case report and literature review

**DOI:** 10.3389/fonc.2025.1589990

**Published:** 2025-05-30

**Authors:** Luying Zhan, Zhiquan Qin, Liu Yang, Yun Chen

**Affiliations:** ^1^ The Second School of Clinical Medicine, Zhejiang Chinese Medical University, Hangzhou, China; ^2^ Cancer Center, Department of Medical Oncology, Zhejiang Provincial People’s Hospital, Affiliated People’s Hospital, Hangzhou Medical College, Hangzhou, China

**Keywords:** case report, immune myocarditis, immune-related adverse events, methyl prednisolone, immune checkpoint inhibitors

## Abstract

Immune checkpoint inhibitor (ICI)-related myocarditis has a low incidence but an extremely high mortality rate, and malignant arrhythmia caused by ICI therapy is even rarer. We report our experience of two patients with myocarditis with conduction system involvement after ICI therapy. Both patients developed myocarditis within a short period after the first ICI dose and they predominantly had conduction bundle involvement with minimal myocardial damage. One patient rapidly progressed from sinus rhythm to complete atrioventricular block, and the patient’s symptoms improved only after pacemaker implantation. The other patient experienced paroxysmal ventricular tachycardia, which was controlled by synchronous corticosteroid therapy. Despite the strong immune side effects caused by ICIs, both patients achieved good clinical outcomes. We also conducted a literature search to explore the pathological mechanisms underpining immune-related myocarditis, as well as discussing the treatment strategies for immune-related myocarditis with conduction system involvement.

## Introduction

Immune-related myocarditis is a serious adverse reaction that occurs during the treatment with immune checkpoint inhibitors (ICIs), with an incidence rate of 0.50%–1.70%and a mortality rate as high as 50% (1). Cases of severe immune-related myocarditis after ICI therapy have a median onset time of 27 days, and 76% of cases of critical myocarditis occur within 6 weeks after the initiation of ICI therapy (2). Immune-related myocarditis mainly manifests as changes on electrocardiogram, chest pain, heart failure, and myocardial infarction, amongst others (3). Previous studies have reported rare cases of multiple organ immunotoxicity, particularly severe myocarditis caused by myocardial conduction system involvement, following the use of the short term ICI therapy. The proposed incidence of immune related myocardial injury with conduction system involvement is only 0.12% (4). Although conduction system involvement in immune-related myocarditis is seldom seen, it is an important indicator of a severe and poor prognosis. Patients with combined high-grade atrioventricular block have a significantly elevated risk of sudden cardiac death. In case of myocarditis caused by ICI therapy, researchers typically use corticosteroid shock therapy or a combination of immunosuppressants, such as mycophenolate mofetil, infliximab, anti-thymocyte globulin, or abatacepta. However, the corticosteroid dosage and treatment strategy have not been fully unified. Here, we report two cases of immune-related myocarditis with conduction system involvement as the main manifestation, accompanied by elevated markers of myocardial injury and muscle involvement such as blepharoptosis. We also explore the potential treatment strategies for patients with immune-related myocarditis.

## Case 1

A 71-year-old woman weighing 51 kg presented to the Oncology Department of Zhejiang Provincial People’s Hospital in June 2024 with a chief complaint of acid reflux and belching accompanied by hidden pain in the left upper abdomen for 15 days. The patient had no history of cardiovascular or autoimmune diseases, nor did she have any other accompanying diseases.

**Figure 1:**
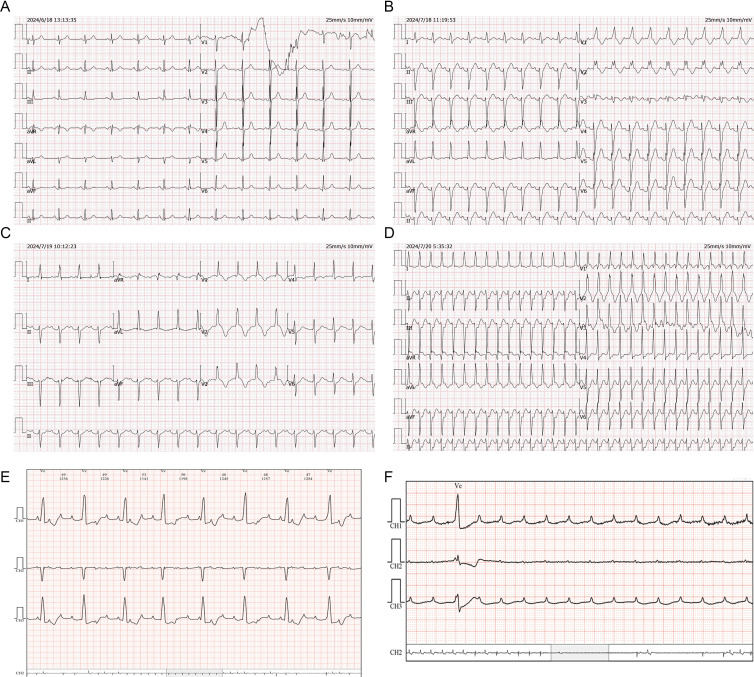
ECG changes of case 1. **(A)** Sinus rhythm. **(B)** Sinus tachycardia, complete right bundle branch block and left anterior branch block. **(C)** Grade I atrioventricular block, ST segment oblique elevation, T wave inversion deepening, and QTc prolongation in anterior septal lead. **(D)** Supraventricular tachycardia. **(E)** Grade III atrioventricular block. **(F)** Ventricular arrest.

**Figure 2:**
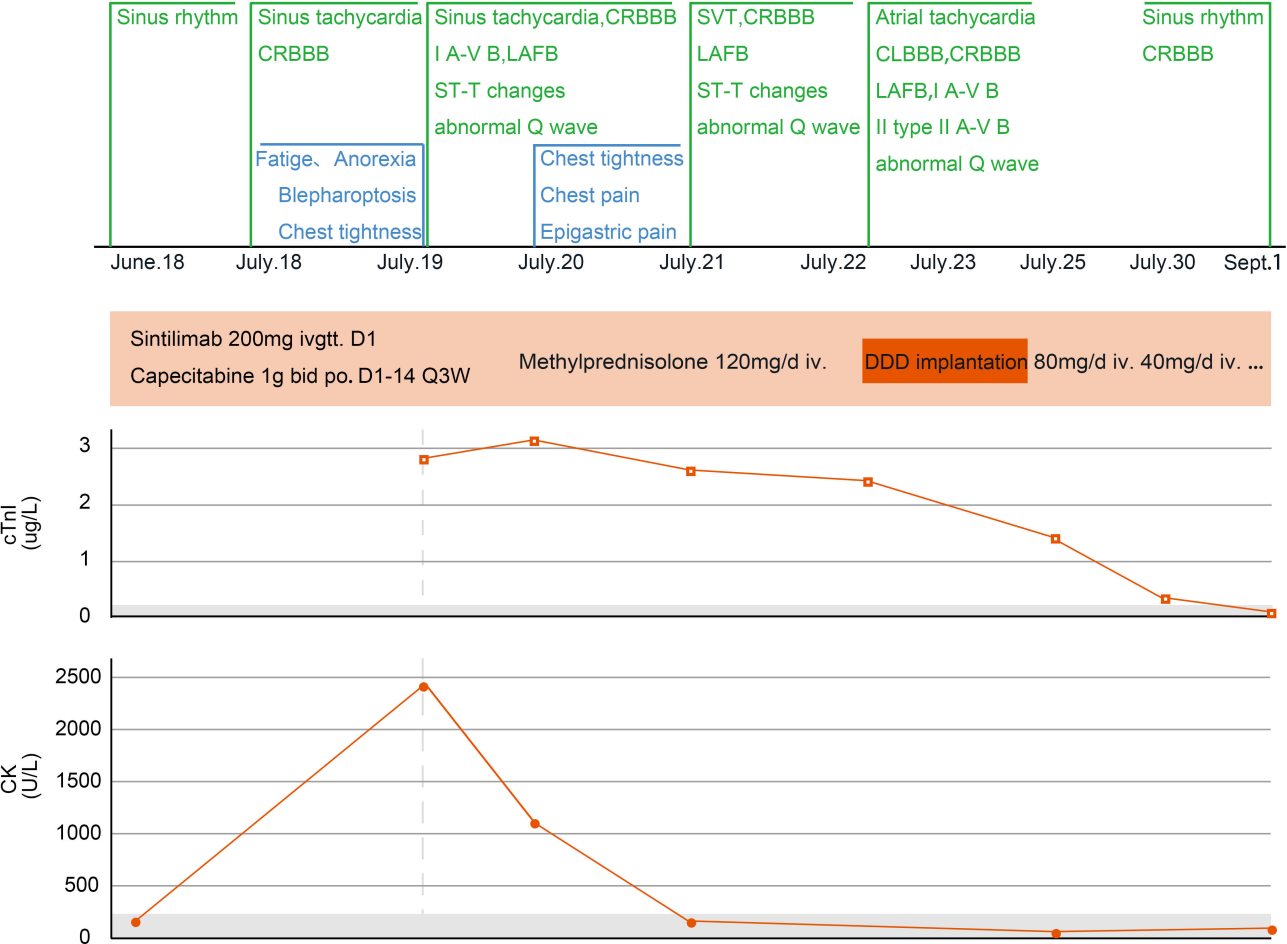
Changes in myocarditis−related indicators of case 1. CRBBB, complete right bundle branch block; I A-V B, first-degree atrioventricular block; LAFB, left anterior fascicular block; SVT, supraventricular tachycardia; CLBBB, complete left bundle branch block; II A-V B, second-degree atrioventricular block; Q3W, every three weeks; DDD, double-chamber pacemaker; cTnI, cardiac troponin I; CK, creatine kinase.

After admission, gastroscopy showed stiffness in the lower segment of the stomach and irregular ulcers near the posterior wall of the greater curvature. Biopsy suggested poorly differentiated adenocarcinoma and partial signet ring cell carcinoma. Immunohistochemistry revealed human epidermal growth factor receptor 2 (0), a programmed death-ligand 1 tumor proportion score of 40% and a combined positive score of 50%, and proficient mismatch repair. Whole-body positron emission tomography/computed tomography (CT) suggested uneven thickening of the gastric wall and increased fluorodeoxyglucose metabolism, suggesting gastric cancer. There was serosal surface involvement, multiple small nodules in the surrounding mesentery, and low metabolic metastases. Initial electrocardiogram showed sinus rhythm (**Figure 1A**), and the myocardial enzyme spectrum showed that the creatine kinase concentration was 46 U/L (normal range 40–200 U/L) (**Figure 2**).

Taking the patient’s age and wishes into consideration, anti-tumor treatment was administered on June 26, 2024 (intravenous infusion of 200 mg sintilimab D1 and 1000 mg oral capecitabine twice a day on days 1–14 and then once every 3 weeks). On day 18 of treatment, the patient experienced discomfort, including fatigue, abdominal distension, and decreased appetite. A gastrointestinal reaction caused by capecitabine was suspected. However, the patient’s symptoms persisted and she gradually developed palpitations, chest tightness, blepharoptosis, and deviation of the angle of the mouth. Therefore, the patient attended our hospital again for treatment.

On July 18, 2024 (day 21 of treatment), electrocardiogram showed sinus tachycardia, complete right bundle branch block, and left anterior branch block (**Figure 1B**). On July 19, 2024 (day 22 of treatment), laboratory tests showed: elevated troponin I (2.823 μg/L; normal value ≤ 0.050 μg/L), B-type natriuretic peptide (184.7 pg/mL; normal value ≤ 160.0 pg/mL), and creatine kinase (2487 U/L; normal range 40–200 U/L). Electrocardiogram revealed new first degree atrioventricular block, anterior septal lead ST segment downward-sloping elevation, deepening of T-wave inversion and QTc prolongation (**Figure 1C**). The total triiodothyronine concentration was 1.43 μg/L (normal range 0.66–1.61 μg/L), total thyroxine concentration was 244.86 μg/L ↑ (normal range 54.40–118.50 μg/L), and the thyroid stimulating hormone concentration was 0.02 mIU/L (normal range 0.34–5.60 mIU/L). The patient was diagnosed with immune myocarditis, arrhythmia, and thyroiditis, and the possibility of concomitant myasthenia gravis was considered based on the patient’s symptoms. Owing to the critical condition of the patient, no neurological diagnostic examination was performed.

On July 19, 2024, intravenous methylprednisolone (120 mg/day) was administered. On July 20, 2024 (day 23 of treatment), follow-up examination showed increase in troponin I to 3.185 μg/L, an increase in B-type natriuretic peptide to 806.5 pg/mL, and a decrease in creatine kinase to 1157 U/L. Frequent supraventricular tachycardia occurred (**Figure 1D**), and methylprednisolone (120 mg/day) was continued. Esmolol and amiodarone were administered intravenously to control the ventricular rate. Myocardial injury-related laboratory markers declined progressively (**Figure 2**), but repeated episodes of supraventricular tachycardia occurred. On July 22, 2024 (day 25 of treatment), second-degree atrioventricular block occurred, which rapidly progressed to third-degree atrioventricular block (**Figure 1E**), with ventricular arrest lasting for up to 9.4 seconds (**Figure 1F**).

Rapid intravenous treatment with isoproterenol and emergency double-chamber permanent pacemaker implantation were performed. After surgery, oral metoprolol sustained-release tablets were administered in combination with intravenous injection of amiodarone to control the patient’s heart rhythm, and methylprednisolone injection (120 mg/day) was continued. The patient’s symptoms, such as chest tightness and fatigue improved, and blepharoptosis and deviation of the angle of the mouth were relieved. From July 19 to July 24, methylprednisolone 120 mg/day intravenous infusion was continued. On July 23, an implantable cardioverter defibrillation was implanted. Starting from July 25, methylprednisolone was changed to 80 mg/day intravenous infusion, and on July 28, it was changed to 40 mg/day intravenous infusion. After, oral methylprednisolone was slowly reduced.

On September 1, 2024, the patient attended our hospital for follow-up. Troponin I was 0.018 μg/L (normal value ≤ 0.050 μg/L), B-type natriuretic peptide was 14.9 pg/mL (normal value ≤ 160.0 pg/mL), and creatine kinase was 146 U/L (normal range 40–200 U/L), Electrocardiogram showed sinus rhythm, complete right bundle branch block, and T-wave changes. Thyroid function was evaluated, and total triiodothyronine was reduced (0.26 μg/L; normal range 0.66–1.61 μg/L), thyroxine was reduced (< 5.50 μg/L; normal range 54.40–118.50 μg/L), and thyroid-stimulating hormone was increased (47.73mIU/L; normal range 0.34–5.60mIU/L). Considering hypothyroidism, oral levothyroxine sodium supplementation (50 μg/day) was administered. On September 2, 2024, a reexamination by abdominal contrast-enhanced CT showed a reduction in the size of the gastric lesion compared with the previous evaluation. As of September 3, 2024, the patient’s condition was stable, and the quality of life was good. Methylprednisolone was discontinued, and during regular follow-ups, anti-tumor treatment has not been administered according to the patient’s wishes.

## Case 2

**Figure 3:**
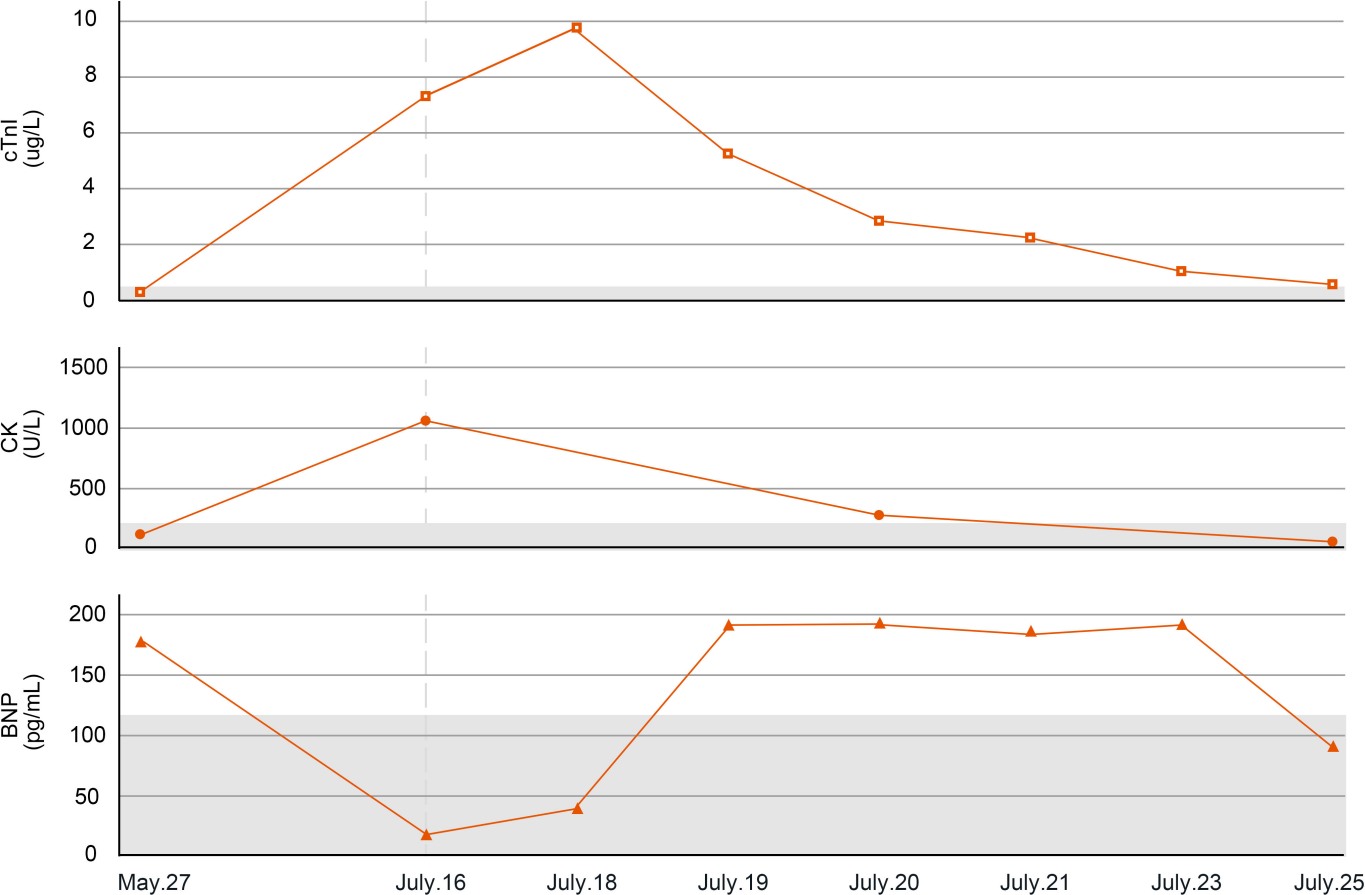
Changes in myocarditis-related indicators of case 2. cTnI, cardiac troponin I; CK, creatine kinase; BNP, B-type natriuretic peptide.

A 64-year-old male weighing 56.5 kg attended the Oncology Department of Zhejiang Provincial People’s Hospital in May 2022 with a chief complaint of cough with lower back pain for 1 month and a diagnosis of lung cancer for 1 day. The patient had no history of cardiovascular disease, autoimmune disease, or other accompanying diseases. Chest CT performed at the local hospital showed a space-occupying lesion in the lower left hilum with obstructive inflammation, as well as multiple slightly enlarged lymph nodes in the left hilum and mediastinum. Pathology suggested squamous cell carcinoma. Electrocardiogram showed sinus bradycardia with left ventricular high voltage, and the myocardial enzyme spectrum showed a troponin I concentration of 0.006 μg/L, B-type natriuretic peptide concentration of 172.5 pg/mL, and a creatine kinase concentration of 99 U/L (**Figure 3**).

Considering the patient’s wishes, two cycles of anti-tumor treatment were administered on May 31, 2022, and June 23, 2022, including intravenous infusion of 200 mg sintilimab D1, intravenous infusion of 100 mg albumin-bound paclitaxel D5, and intravenous infusion of 0.35 g carboplatin D1 every 3 weeks. On day 42 of the initial treatment, the patient experienced blurred vision and diplopia in both eyes.

**Figure 4:**
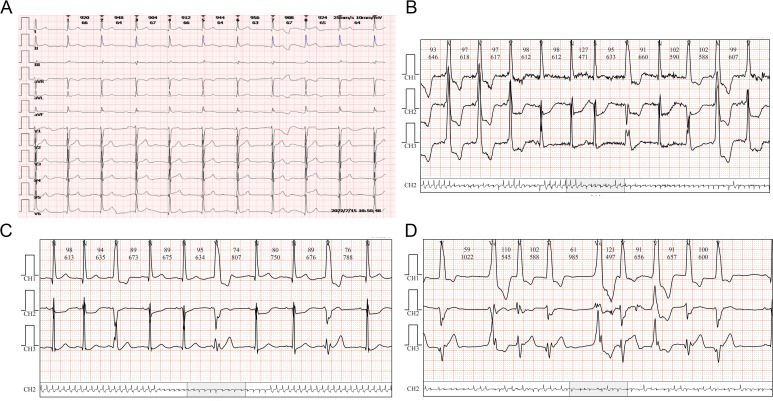
ECG changes of case 2. **(A)** Sinus rhythm. **(B)** Atrial premature beats. **(C)** Multi-source ventricular premature beats. **(D)** Paroxysmal ventricular tachycardia and accelerated ventricular escape.

On July 15, 2022 (day 45 of treatment), the patient attend our hospital for treatment, and electrocardiogram showed sinus rhythm (**Figure 4A**). On July 16, 2022 (day 46 of treatment), laboratory tests showed normal thyroid function, with troponin I elevation (7.805 μg/L), B-type natriuretic peptide reduction (19.5 pg/mL), and creatine kinase elevation (1068 U/L). At that time, the patient had no chest tightness or dyspnea and was observed with dynamic monitoring of troponin levels. On July 18, 2022 (day 48 of treatment), the patient complained of slight chest tightness, which was not severe, and he still had blurred vision. Reexamination showed an increase in troponin I to 9.807 μg/L and B-type natriuretic peptide to 48.7 pg/mL. Echocardiography indicated decreased left ventricular compliance and normal systolic function. Ophthalmic examination showed possible paralysis of the right inferior rectus muscle. The patient was diagnosed with immune myocarditis and possible concomitant myositis.

On July 18, 2022, methylprednisolone was administered intravenously at a dose of 40 mg/day. On July 19, 2022 (day 49 of treatment), a follow-up examination showed a slight decrease in troponin I (5.688 μg/L) and a significant increase in B-type natriuretic peptide (184.8 pg/mL). Methylprednisolone was administered intravenously at a dose of 80 mg/day. On July 20, 2022 (day 50 of treatment), reexamination showed a significant decrease in troponin I to 2.746 μg/L, B-type natriuretic peptide of 189.4 pg/mL, and creatine kinase of 347 U/L. At the same time, dynamic electrocardiogram showed atrial premature beats (**Figure 4B**), multi-source ventricular premature beats (**Figure 4C**), paroxysmal ventricular tachycardia and accelerated ventricular escape (**Figure 4D**). Intravenous methylprednisolone (80 mg/day) was continued, followed by intravenous esmolol and amiodarone to control the ventricular rate. Subsequently, the laboratory indicators showed a sustained decline. On July 21, 2022 (day 51 of treatment), troponin I was 2.075 μg/L and B-type natriuretic peptide was 182.6 pg/mL. Intravenous methylprednisolone was reduced to 40 mg/day. On July 23, 2022 (day 53 of treatment), troponin I was 1.103 μg/L and B-type natriuretic peptide was 188.1 pg/mL. On July 25, 2022 (day 55 of treatment), troponin I was 0.484 μg/L, B-type natriuretic peptide was 93.7 pg/mL, and creatine kinase was 60 U/L.

On July 26, 2022 (day 56 of treatment), the patient had no chest tightness or dyspnea, and blepharoptosis had improved. The patient requested to be discharged and switched to oral methylprednisolone for slow reduction. Unfortunately, the patient did not return to the hospital for follow-up evaluation.

## Discussion

Mechanistically, ICIs enhance anti-tumor immunity by activating T cells and relieving immune suppression. However, this can trigger autoimmune responses by increasing autoantibodies and cytokines, leading to immune-related adverse events. Most patients with immune-related myocarditis present with myocardial injury and cardiac dysfunction, while conduction system involvement causing malignant arrhythmia is far less common. The onset of critical myocarditis tends to occurred within 6 weeks after the initiation of ICIs, which is similar to the two cases we reported in the present study.

The mechanism by which ICIs cause arrhythmia is largely unknown. Johnson et al. (5) observed that there are high-frequency T cell coreceptor sequences in the infiltration of myocardium, skeletal muscle, and tumors, suggesting that tumor cells and myocytes (conduction cells) may share certain antigens. Activated T cells may mistakenly identify certain antigens in the cardiac conduction system as tumor antigens, leading to cross immune attacks. Johnson et al. also observed necrosis and infiltration of CD4^+^, CD8^+^T cells and macrophages in the myocardium and conduction system of patients with melanoma treated with nivolumab plus ipilimumab. Similarly, Zotova et al. (6) also observed that ICI-mediated lymphocytes can infiltrate the cardiac conduction system (sinoatrial node, atrioventricular node), directly destroying conduction cells. Additional basic and translational research is urgently needed to fully understand the pathophysiology of ICI-induced arrhythmia, which will help to develop treatment strategies specifically targeting this population.

For ICI-related myocarditis, the dosage and treatment strategy of conventional corticosteroid shock therapy have not been fully unified. The Chinese Society of Clinical Oncology and the National Comprehensive Cancer Network guidelines recommend that patients with severe myocarditis should immediately receive methylprednisolone shock therapy at a dose of 500–1000 mg/day for 3–5 days, followed by gradual reduction. However for patients with minimal myocardial damage, 1–4 mg/kg/day methylprednisolone can be administered continuously for 3–5 days, gradually reducing the dose. The initial presentation of the patients in present case was mainly mild elevation of markers of myocardial injury, without indications for corticosteroid shock therapy. Therefore, methylprednisolone was administered at a conventional dose, and good laboratory indicator control was ultimately achieved.

To further explore the practical evidence supporting the glucocorticoid dosage, we searched PubMed identify reports of patients with solid tumors with immune-related myocarditis, which mainly manifested as arrhythmia caused by ICIs. The search included studies published from January 1, 2016, to December 1, 2024, and the following information was extracted: author, year, age, sex, primary lesion, ICIs, main manifestations, other adverse reactions, corticosteroid dosage, and prognosis. Among the 19 cases reporting the corticosteroid dosage (**Table 1**), all patients showed electrocardiogram changes. Three cases presented mainly with ventricular arrhythmia, whiling the remaining 16 patients presented mainly with complete atrioventricular blockade. Most cases exhibited changes in myocardial injury markers, decreased left ventricular ejection fraction, and neuromuscular involvement. In terms of the strategy of corticosteroid use, 10 cases received corticosteroids at doses used for shock, seven received corticosteroids at conventional doses, and two were not treated with corticosteroids. The implantation of temporary or permanent pacemakers did not significantly affect the prognosis of the patients treated with corticosteroids at doses used for shock. Among the patients treated with conventional doses of corticosteroids, three achieved symptom improvement, all of whom had temporary or permanent pacemakers implanted. The remaining patients died as a result of malignant arrhythmia or hemodynamic instability. Of the remaining two patients who did not receive corticosteroid treatment, one achieved symptom improvement after pacemaker implantation, and the other was controlled by treatment with Impella and extracorporeal membrane oxygenation. Another retrospective study conducted in Italy showed that no differences in outcomes were observed among patients with immune-mediated myocarditis treated with different maximum daily doses of corticosteroids (7), but the study did not further analyze the subgroup of patients with arrhythmia as the main manifestation (47/84). This suggests that for patients with immune-related myocarditis involving the conduction system, the appropriate dosage of corticosteroids requires further exploration, and early pacemaker implantation may be more important to improve patient prognosis. A previous literature review evaluating 30 patients with secondary conduction disorders caused by ICIs also emphasized the importance of temporary pacemaker implantation (8).

**Table 1:** Demographic parameters, presenting features, maximum daily dose of corticosteroids and outcomes of included cases.

Author	Year	Sex	Age	Indication	ICIs	Arrhythmia	EF	Other System involvement	Maximum daily dose of Corticosteroids	Pacemaker	Other Treatment	In-hospital outcome	Reference
Johnson	2016	F	65	Melanoma	Nivolumab+ipilimumab	CAB	–	Myositis Rhabdomyolysis	1mg/kg/d			Death	(5)
Johnson	2016	M	63	Melanoma	Nivolumab+ipilimumab	CAB	↓	Myositis	1g/d	ICD	Infliximab	Death	(5)
Behling	2017	M	63	Melanoma	Nivolumab	CAB		Myositis Rhabdomyolysis Renal insufficiency	1.5mg/kg/d	ICD		Death	(14)
Khan	2020	F	67	Lung	Pembrolizumab	CAB	–			ICD		Alive	(15)
Prevel	2020	M	80	Lung	Nivolumab	CAB		Rhabdomyolysis	1mg/kg/d	ICD		Death	(16)
Stein-Merlob	2021	F	60	Colon	Nivolumab	VT	↓	Myositis Hepatic insufficiency Colitis		Impella+ECMO		Alive	(17)
Nguyen	2021	M	25	Thymoma	Pembrolizumab	CAB	↓		1g/d		MMF+Ruxolitinib+ Abatacept	Alive	(18)
Jesperson	2021	M	57	Renal	Nivolumab+ipilimumab	CAB	↓	Myositis Hepatic insufficiency	1g/d	ICD	MMF+ Abatacept	Alive	(19)
Portolés-Hernández	2021	F	48	Thymoma	Pembrolizumab	CAB	↓	Myositis Hepatic and renal insufficiency	1g/d	ICD	Infliximab	Death	(20)
Ye	2022	M	71	Cholangiocarcinoma	Teriprizumab	CAB	–		200mg/d	ICD		Alive	(21)
Zhang	2022	F	68	Thymoma	Camrelizumab	CAB	–	Myositis Hepatic insufficiency	1g/d	ICD	IVIG	Death	(22)
Su	2022	F	80	Intraosseous squamous cell carcinoma	Pembrolizumab	CAB	–		1mg/d	ICD		Alive	(8)
Wang	2023	F	45	Thymoma	Sintilimab	VF	–	Myositis	120mg/d		IVIG+PE	Death	(23)
Davis	2023	F	60+	Lung	Durvalumab	VPB	↓		1g/d	ICD		Alive	(24)
Diaz-Rodriguez	2023	M	80	Liver	Durvalumab+tremelimumab	CAB	↓	Myositis Hepatic insufficiency	1g/d	ICD	MMF	Death	(25)
Zhong	2023	M	55	Thymoma	PD-1	CAB		Myositis	1g/d	ICD	MMG+IVIG	Alive	(26)
Li	2024	F	66	Breast	Sintilimab	VT		Hepatic and renal insufficiency	500g/d		IVIG	Alive	(27)
Wang	2024	M	72	Gastric	Sintilimab	CAB		Myositis	200mg/d	ICD	IVIG	Alive	(28)
Wang	2024	M	60	Lung	Pembrolizumab	CAB	–		1g/d	ICD	IVIG	Alive	(29)

CAB, Complete Atrioventricular Block; VT, Ventricular Tachycardia; VF, Ventricular Fibrillation; ICD, Implantable Cardioverter Defibrillator; MMF, Mycophenolate Mofetil; IVIG, Intravenous Immunoglobulin; PE, Plasma Exchange.

Notably, Case 1 in the present study achieved good tumor control simultaneously with the intense immune side effects induced by sintilimab, suggesting a possible relationship between the degree of immune side effects and the degree of tumor control. The occurrence of adverse drug reactions is usually not conducive to the effectiveness of disease treatment. However, for patients undergoing immunotherapy, the occurrence of immune-related adverse reactions may indicate that the immune system has been fully activated, potentially leading to better clinical efficacy (9). Patients who experience severe immune-related adverse reactions should have higher T-cell activity than patients with mild immune-related adverse reactions. Multiple studies have reported that patients who develop immune-related adverse reactions after ICI therapy achieve significantly higher clinical efficacy than those who do not develop immune-related adverse reactions (10, 11). A study involving 139 patients with metastatic melanoma treated with ipilimumab showed that all patients who achieved a complete response had more severe immune-related adverse reactions (12). In another retrospective analysis, the overall survival of patients with non-severe immune-related adverse reactions, severe immune-related adverse reactions, and no immune-related adverse reactions was 1.1, 0.9, and 0.6 years, respectively, suggesting a possible relationship between the severity of immune-related adverse reactions and the efficacy of ICI therapy (13). Of course, the incidence of adverse reactions related to different tissues and organs varies among different types of cancer. This may be influenced by multiple factors, such as the tumor microenvironment, immune infiltration, the adaptive immune response, and new antigen formation. The patients we reported in the present study experienced strong adverse reactions, but they also achieved good lesion control. However, large-scale studies are needed to explore the relationship between the degree of immune-related adverse reactions and the clinical efficacy of treatment.

## Conclusion

We reported two rare cases of immune-related myocarditis with conduction system involvement as the main manifestation. Unlike previously reported cases, the patients in the present study achieved good outcomes after treatment with conventional doses of corticosteroids and pacemaker implantation. These findings suggest that we should focus on the impact of ICIs on the myocardial conduction system during the management of immune side effects. Further exploration is needed to determine the optimal glucocorticoid treatment strategy.

## Data Availability

The original contributions presented in the study are included in the article/supplementary material. Further inquiries can be directed to the corresponding authors.
